# Diatoms from Brazil: the taxa recorded by Christian Gottfried Ehrenberg

**DOI:** 10.3897/phytokeys.18.3653

**Published:** 2012-11-19

**Authors:** Weliton José da Silva, Regine Jahn, Mariângela Menezes

**Affiliations:** 1 Labfico, Departamento de Botânica, Museu Nacional, Universidade Federal do Rio de Janeiro, Rio de Janeiro, Brazil– Programa de Pós-graduação em Ciências Biológicas (Botânica); 2 Botanischer Garten und Botanisches Museum Berlin-Dahlem, Freie Universität Berlin; 3 Labfico, Departamento de Botânica, Museu Nacional, Universidade Federal do Rio de Janeiro, Rio de Janeiro, Brazil

**Keywords:** Lectotypification, nomenclatural status, Ehrenberg collection, *Eunotia*, *Pinnularia*, *Terpsinoe*

## Abstract

The flora of diatoms from Brazil has been studied by several authors from the beginning of the 19^th^ up to now. Some of the old lists and descriptions are unknown or have been ignored by Brazilian researchers and the situation of the names cited was not assessed. Here we compiled a list of 101 taxa of diatoms from Brazil registered by Christian Gottfried Ehrenberg during the 19^th^ century. We checked the current nomenclatural status of those taxa and lectotypified species from Brazil described by this author. For this, we accessed the Ehrenberg collection in the Museum für Naturkunde, Berlin, Germany, where 11 samples from Brazil studied by Ehrenberg are housed and published in different papers. Using these samples, we found 101 taxa (specific and infraspecific) published by Ehrenberg from Brazil. Five species (*Eunotia bidens* Ehrenb., *Eunotia depressa* Ehrenb., *Eunotia elephas* Ehrenb., *Pinnularia microstauron* Ehrenb., and *Terpsinoe brasiliensis* Ehrenb.) were new descriptions and were lectotypified here. The other species cited for Brazil were described initially from other places. However, 23 names were invalid and one illegitimate.

## Introduction

The 19^th^ century was very important regarding the description of the biodiversity of algae and protists. At the first haft of that century, material from different parts around the world was analyzed by researchers from Europe and is today deposited in institutions on this continent. Christian Gottfried Ehrenberg was one of these early researchers (Lazarus and Jahn 1998). His collection comprises material from Africa, America, Europe, Asia and Oceania. However, the collection was mostly unavailable until 1990 (Lazarus and Jahn 1998). This inaccessibility and the absence of designated nomenclatural types of many taxa described by him, resulted in unclear taxonomic concepts of a number of these species.”

Brazil is among the regions from South America whose diatom flora was studied by Ehrenberg. Samples from the States of Amazonas, Rio de Janeiro, São Paulo, Minas Gerais and Santa Catharina were analyzed by Ehrenberg and the names of some species were published in five publications (Ehrenberg 1839, 1841, 1843, 1851, 1854). Several of the taxa names published by him are in apparent disuse, and the current nomenclature of many of them is not known. This is true for the entire list of Ehrenberg’s names. The validation of these names is guided by the ICZN (International Code of Zoological Nomenclature), as stated by Article 45.4 of the International Code of Botanical Nomenclature (ICBN) (McNeill et al. 2006), since diatoms were considered by Ehrenberg as polygastric animals.

Recently, Brazil is among the countries that have employed efforts to repatriate data of plants collected by foreign researchers during 18^th^, 19^th^ and 20^th^ centuries. Such efforts have resulted positively in the creation of virtual herbaria (Zappi et al. 2012) and in the cataloguing of the flora thus increasing lists of species recorded in all its territory (Forzza et al. 2012). Several European collections have diatom material from Brazil (e.g. Ehrenberg, Grunow, Hustedt, Tempère & Peragallo, Krasske) which is still mostly unexplored. Such collections are very important for taxonomic studies which are the base to the knowledge of the biodiversity and, consequently, to the knowledge of tropical aquatic systems. Thus, this is the first work that deals of repatriation of data about algae, specifically diatoms.

The aim of this study was to compile a list of taxa of diatoms from Brazil registered by Ehrenberg, to check the current nomenclatural status of those taxa, and to lectotypify specific and infraspecific taxa.

## Material and methods

The Ehrenberg Collection (i.e., preparations, drawings and publications) was accessed at BHUPM (Museum für Naturkunde, Leibniz-Institut für Biodiversität- und Evolutionsforschung an der Humboldt Universität zu Berlin), where it is deposited and recorded under the following numbers of cases (Kästen) and card folders (Bücher): K. 18 B. 3-5, K. 19 B. 9, K. 52 B. 12, K. 53 B. 10; Jahn and Kusber (2004) have transcribed this for the use in databases; the material thus includes the following micastrips: 180301-180316, 180401-180416, 180501-180516, 190901-190916, 521201-521216, 531001-531016. The data of the samples (i.e., original number of sample, locality, collector (leg.), date of sampling, study on which the analysis of such samples were published) are compiled in Table 1. The original number of sample is maintained by BHUPM as current control number.

**Table 1.** Data about samples of diatoms from Brazil analyzed by Ehrenberg.<br/>

**Original sample**	**Original name of the locality**	**Current name of the locality**	**Coordinates**		**Collector**	**Date of sampling**	**Micastrips**	**Extra data of the samples**	**Reference**
1085	Ega	Tefé, Amazonas State	3°20'29.87"S, 64°43'33.39"W		Eduard Friedrich Poeppig	Between 1831 and 1832	180313-180316	Subaerial, moss *Hypnum acuminulatum*	Ehrenberg (1854)
*	Ega-See	Tefé Lake, Tefé, Amazonas State	3°20'29.87"S, 64°43'33.39"W		Eduard Friedrich Poeppig	Between 1831 and 1832	180416	Periphytic, under *Oscillaria* sp.	Ehrenberg (1854)
1104	Praya de Sernambatyba	Barra Beach, Rio de Janeiro, Rio de Janeiro	23°0'35.56"S, 43°22'8.97"W		Karl Sigismund Kunth	Between 1829 and 1841	180501-180502	Root of *Eriocaulon mosestum*	Ehrenberg (1843, 1854)
1105	Santo Antonio do Monte	Santo Antônio do Monte, Minas Gerais State	20°4'50.27"S, 45°17'55.27"W		Karl Sigismund Kunth	Between 1829 and 1841	180503-180504	Humus attached to Gramineae	Ehrenberg (1843, 1854)
1106	São Paulo	São Paulo, coast	24°5'31.91"S, 46°43'6.76"W		Édouard Louis Chavannes	1846*	180507-180508	Soil	Ehrenberg (1854)
1099	Rio Conigo in Rio de Janeiro A	Cônego River, Nova Friburgo, Rio de Janeiro State	22°19'33.03"S, 42°33'5.48"W		Hermann Encke	07th April 1850	180401-180406	Sediment	Ehrenberg (1851, 1854)
1100	Rio Conigo in Rio de Janeiro B	Cônego River, Nova Friburgo, Rio de Janeiro State	22°19'33.03"S, 42°33'5.48"W		Hermann Encke	07th April 1850	180407-180408	Sediment	Ehrenberg (1851, 1854)
1101	Insel St. Catharina, Rio de Lauro	Santa Catarina	26°28'24.01"S, 48°57'9.32"W		Carl Pabst	1850	180409-180410	Subaerial, moss *Hypnum* sp.	Ehrenberg (1854)
1102	Insel St. Catharina, Rio Concescao	Santa Catarina	26°28'24.01"S, 48°57'9.32"W		Carl Pabst	1850	180411-180412	Subaerial, moss *Hypnum loxense*	Ehrenberg (1854)
1103	Insel St. Catharina, Barre des Itajahi	Santa Catharina	26°28'24.01"S, 48°57'9.32"W		Carl Pabst	November and December 1846	180413	Subaerial, moss *Sphagnum pulchricoma*	Ehrenberg (1854)
1087	Sumpfboden, Amazonas, Coari	Coari, Amazonas State	4°5'13.84"S, 63°8'40.87"W		Carl Friedrich Philipp von Martius		180301-180308		Ehrenberg (1839, 1841, 1843)

* The Sample Index did not provide the number of the respective sample

To verify the nomenclature of some names, beside Ehrenberg’s original publications, we consulted Index Nominum Algarum (2012), Algaebase (Guiry and Guiry 2012) and Algaterra (Jahn and Kusber 2012).

The occurrence of the taxa cited for Brazil but originally described from other places than Brazil is provided in this study by the number of original samples (Table 1), except the sample from Tefé Lake (Ega-See) for which the Sample Index did not provide the number of the original sample and which will be referred to as Tefé Lake.

## Results

### A brief history about the samples from Brazil

Ehrenberg never went to Brazil. However, he received samples from different important persons of the history of such country. The main was Carl Friedrich Philipp von Martius (1794–1868), a researcher of the Brazilian flora, who sent samples from Coari (Amazonas State) to Ehrenberg (1839, 1841, 1843). Karl Sigismund Kunth (1788–1850), Eduard Friedrich Poeppig (1788–1868), Édouard Louis Chavannes (1805–1861) were other naturalists that travelled though South America, including Brazil, and sent samples from Rio de Janeiro and Minas Gerais States, Amazonas State and São Paulo State, respectively (Ehrenberg 1843, 1854). On the other hand, the samples from Santa Catarina State were collected by Carl Pabst (1825–1863) who worked as a land surveyor in the old Dona Francisca Colonie (today Joinville city) and was an assistant of the botanist Franz Gustav Straube (1802–1853) (Straube 1992). However, these samples were sent to Ehrenberg by Carl Alfred Müller (1855–1907) (Ehrenberg 1854), aGerman bryologist.

**Figure 1.**
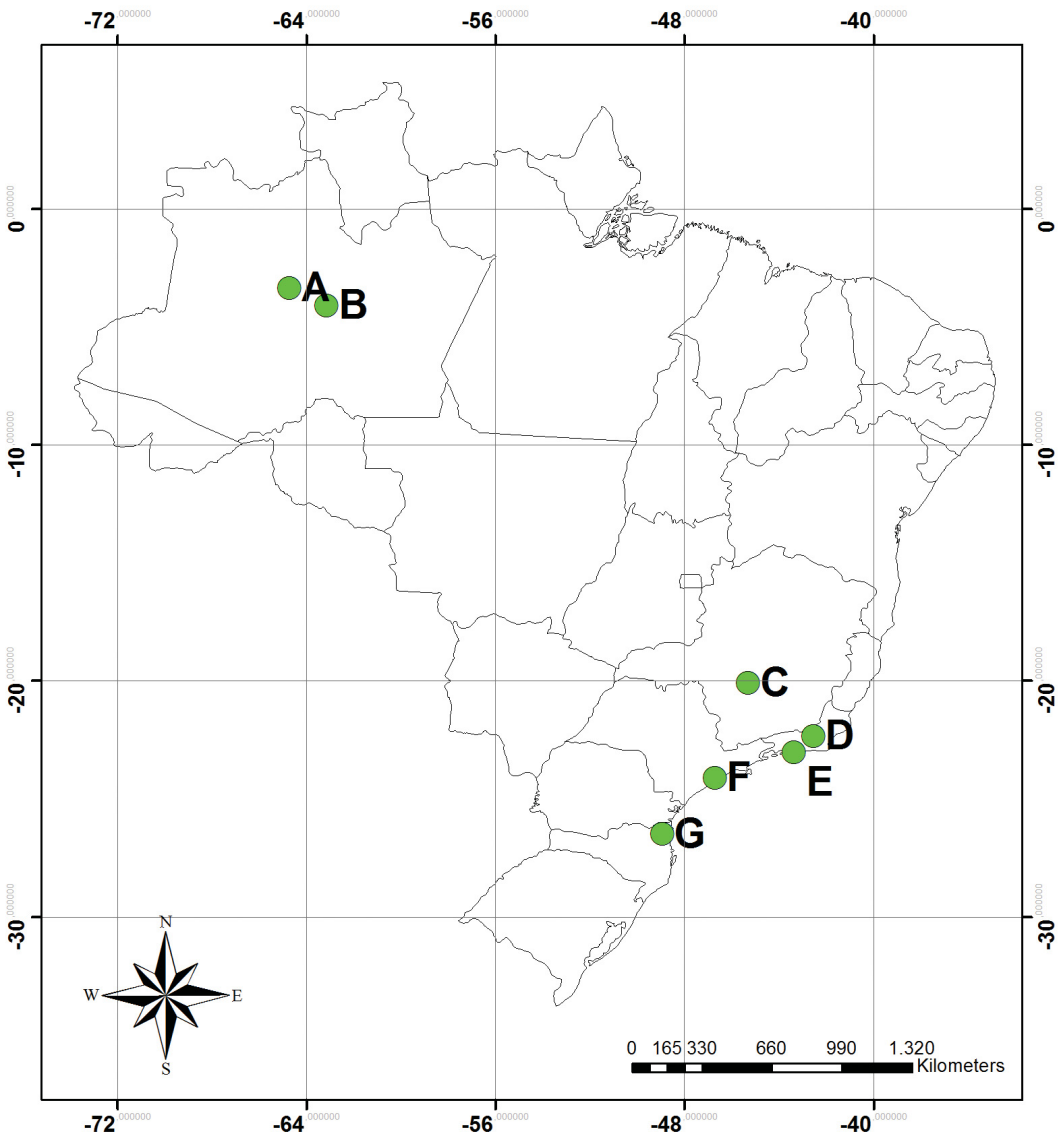
Localities of the samples from Brazil studied by Ehrenberg (1841, 1843, 1851, 1854); **A** Ega (Tefé), Amazonas **B** Coari, Amazonas **C** Santo Antônio do Monte, Minas Gerais **D** Rio Conigo (Cônego River), Nova Friburgo, Rio de Janeiro **E** Praya de Sernambatyba (Barra Beach), Rio de Janeiro **F** São Paulo **G** Santa Catarina.

**Figure 2–5.**
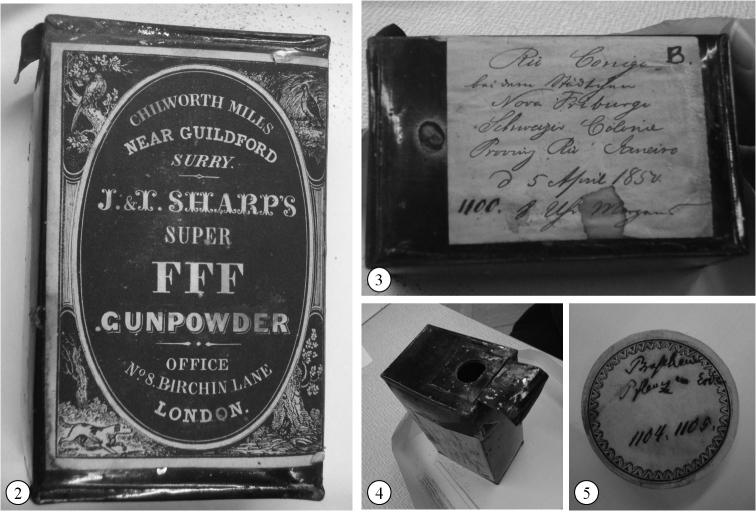
General storage of samples from Brazil in Ehrenberg Collection, Museum für Naturkunde **2–4** Gunpowder boxes **2–3** Lateral view **4** View from the top **5** Medicine box

Twelve samples from five States from Brazil were sent to Ehrenberg (Fig. 1), but only eleven were cited by him in his studies, and one sample was not catalogued in the Sample Index (i.e., Tefé Lake, vide Table 1). The samples were stored in powder boxes (Figs 2–4) or old medicine boxes (Fig. 5) and are kept in the Museum für Naturkunde in a very good state. As well as the other Ehrenberg samples, the preparations of material from Brazil were made using mica discs, embedded with Canada Balsam (Lazarus and Jahn 1998).

From these samples, we cataloged 101 taxa (specific and infraspecific) published by Ehrenberg from Brazil, of which five were new descriptions, 72 whose first descriptions were made from other places than Brazil, and 23 were invalid and one illegitimate name.

The list of all taxon names is given below, and the species described initially from Brazil are here lectotypified.

### Types of valid and legitimate names (available proposals) firstly described from Brazil

#### Eunotia bidens

Ehrenb., Abh. K. Akad. Wiss. Berlin, Physik. Kl., 1841: 413, 1843.

http://species-id.net/wiki/Eunotia_bidens

Figs 6–7

##### Lectotype (designated here).

**Figures 6–14.**
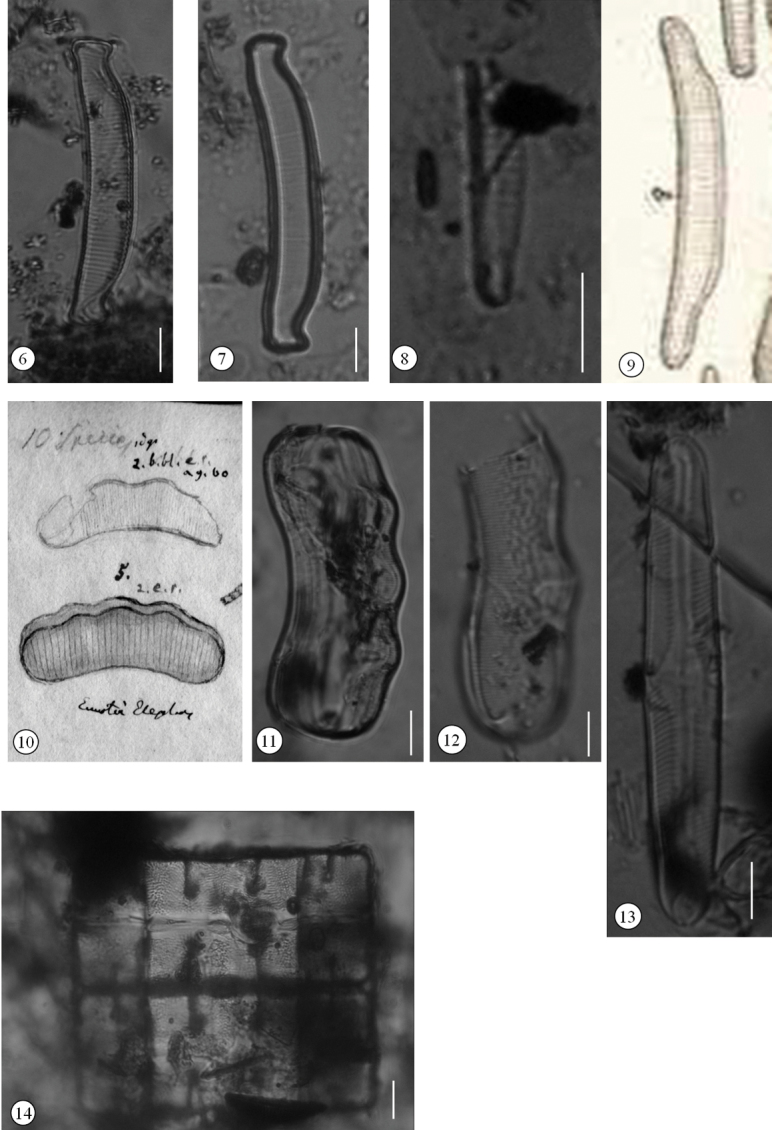
Diatoms from Brazil published by Christian Gottfried Ehrenberg **6–7** *Eunotia bidens* Ehrenb **6** Lectotype, preparation 180404b, marked with yellow (g) ring, from sample 1099 “Rio Conigo in Rio de Janeiro”, Ehrenberg Collection, in BHUPM (Museum für Naturkunde) **7** Another specimen of *Eunotia bidens* found in preparation 180405a, marked with yellow (g) ring **8–9** *Eunotia depressa* Ehrenb **8** Lectotype, preparation 180403a, marked with yellow (g) ring, from sample 1099, “Rio Conigo in Rio de Janeiro”, Ehrenberg Collection, in BHUPM **9** *Eunotia depressa* provided by Ehrenberg (1843, Tafel I, Fig IV: 6b) **10–12** *Eunotia elephas* Ehrenb **10** Part of Ehrenberg’s drawing sheet No. 2050 from “Praya de Sernambatyba” (Barra Beach) **11** Lectotypus, preparation 180502e, marked with red (r) ring, from sample 1104 “Praya Senambatyba, Rio de Janeiro”, Ehrenberg Collection, BHUPM **12** Another specimen of *Eunotia elephas* found in preparation 180502e, marked with red (r) **13** *Pinnularia microstauron* (Ehrenb.) Cleve, lectotype, preparation 180502d, marked with red (r) ring, from sample 1104, “Praya de Sernambatyba, Rio de Janeiro”, Ehrenberg Collection, in BHUPM **14** *Terpsinoe brasiliensis* Ehrenb., lectotype, preparation 180411a, marked with blue (bl) ring, from sample 1102, “Rio Consescao der Insel St. Catharina”, in BHUPM; Scale bars: 10 µm

Specimen in preparation 180404b, marked with yellow (g) ring, from sample 1099 “Rio Conigo in Rio de Janeiro”, Ehrenberg Collection, in BHUPM (Museum für Naturkunde) (Fig. 6).

##### Locality of the lectotype.

Rio Conigo [Cônego River], Nova Friburgo, Rio de Janeiro, Brazil.

##### Locality of the syntypes.

New-York, Andower “Conn.” (Connecticut)

##### Original description.

“*striata, ventre plano, dorso convexo medio exciso (bidentato), apicibus dilatatis truncatis. =* E. diodon *apicibus truncatis*.”

Ehrenberg (1843, p. 373) cited the original locality as Brazil and USA (New York and Andower, Connecticut). In the material from New York (preparations 250401-250408, 260101-260112 and 260301-260308)we did not find any reference to *Eunotia bidens*, even under the name *Himantidium* that Ehrenberg (1843) related to the taxon on page 373. On the other hand, in the material from Andower (preparations 260201-260208) we found a reference to *Eunotia bidens* in the preparation 260205d, marked with a blue ring, but it was not in a good condition to be photographed. Therefore, we chose the material from Brazil as lectotype.

#### Eunotia depressa

Ehrenb., Abh. K. Akad. Wiss. Berlin, Physik. Kl., 1841: 413, Tafel I, Fig IV: 6b, Tafel IV, Fig I: 12, 1843

http://species-id.net/wiki/Eunotia_depressa

Fig 8–9

##### Lectotype (designated here).

Specimen in preparation 180403a, marked with yellow (g) ring , from sample 1099, “Rio Conigo in Rio de Janeiro”, Ehrenberg Collection, in BHUPM (Museum für Naturkunde) (Fig 8).

##### Locality of the lectotype.

Rio Conigo [Cônego River], Nova Friburgo, Rio de Janeiro, Brazil.

##### Locality of the other syntype.

“New York?”

##### Original description.

“*striata, anguste linearis, ventre plano aut leviter concavo, dorso depresso plano, prope apices rotundatos subito attenuato*.”

Ehrenberg (1843, p. 373) defined that *Eunotia depressa* occurred in Brazil and possibly in USA (New York). We verified the preparations from New York (K: 25, B: 4; K: 26, B: 1, 3) but did not find any citation of *Eunotia depressa*. Therefore, we defined the lectotype from the material from Brazil.

#### Eunotia elephas

Ehrenb., Abh. K. Akad. Wiss. Berlin, Physik. Kl., 1841: 414, Tafel I, Fig IV: 5, 1843.

http://species-id.net/wiki/Eunotia_elephas

Figs 10–12

##### Lectotype (designated here).

Specimen in preparation 180502e, marked with red (r) ring, from sample 1104 “Praya Senambatyba, Rio de Janeiro”, Ehrenberg Collection, BHUPM (Museum für Naturkunde) (Fig. 11).

##### Locality of the lectotype.

“Praya de Sernambatyba”, Rio de Janeiro, Brazil.

##### Original description.

“*striata, latissima, curva, apicibus late rotundatis, dorso, tridentato*.”

#### Pinnularia microstauron

(Ehrenb.) Cleve, Acta Soc. Fauna Fl. Fenn. 8(2): p. 28. 1891

http://species-id.net/wiki/Pinnularia_microstauron

Fig. 13

Stauroptera microstauron Ehrenb., Abh. K. Akad. Berlin, Phys. Kl. 1841: 423, Tafel I, Fig IV: 1, Tafel IV, Fig II: 2, 1843. [Basionym]

##### Lectotype (designated here).

Specimen in preparation 180502d, marked with red (r) ring, from sample 1104, “Praya de Sernambatyba, Rio de Janeiro”, Ehrenberg Collection, in BHUPM (Museum für Naturkunde) (Fig. 13).

##### Locality of the lectotype.

“Praya de Sernambatyba”, Rio de Janeiro, Brazil.

##### Locality of the syntype.

Labrador.

##### Original description.

“*testula styliformis a ventre linearis, lateribus rectis, apicibus arcte constrictis late rotundatis*.”

Krammer (1992, pl. 32, fig. 10) [reproduction of Ehrenberg’s published figure (1843, Tafel I, Fig. IV:1)] defined an “iconotype” for *Pinnularia microstauron*. Since we found the specimen used by Ehrenberg to describe the species, we designated it here as lectotype of the species. *Stauroptera microstauron* was described for two samples from Brazil and Canada (Labrador) (Ehrenberg 1843, p. 387). However, we did not find any mentioning of *Stauroptera microstauron* in the legends of the mica from Labrador (250509 to 250516). Therefore, we defined the lectotype from the material from Brazil.

#### Terpsinoe brasiliensis

Ehrenb., Mikrogeologie, 310, 311, 1854.

http://species-id.net/wiki/Terpsinoe_brasiliensis

Fig. 14

##### Lectotype (designated here).

Specimen in preparation 180411a, marked with blue (bl) ring, from sample 1102, “Rio Consescao der Insel St. Catharina”, in BHUPM (Museum für Naturkunde) (Fig. 14).

##### Locality of the lectotype.

“Rio de Concescao, Insula St. Catharina, Brasilien”.

##### Original diagnosis.

“*mit sehr kleinen Notenzeichen*”.

*Terpsinoe brasiliensis* was published the first time by Ehrenberg in his book Mikrogeologie (1854). Several taxa of diatom published by Ehrenberg in this work are considered unavailable (invalid) according the Article 12 of ICZN (Ride et al. 1999) due to absence of a description, definition and indication of any illustration. This is not the case for *Terpsinoe brasiliensis* and, maybe, could be the only case, in which Ehrenberg provided the following description “…*und* Terpsinoë brasiliensis, *mit sehr kleinen Notenzeichen*…” [and *Terpsinoe brasiliensis*, with very short musical notes]. This short description is considered to be enough by us, as well as several other descriptions published long ago by other authors (e.g., Agardh 1827).

### First descriptions from other localities than Brazil

We provide a list of taxa cited by Ehrenberg as occurring in Brazil. This list is names based and has not been checked with respect to current taxonomy. Authors of combinations have been checked.

***Amphitetras antediluviana*** Ehrenb., Abh. Königl. Akad. Wiss. Berlin, 1839: 142, 1840. [cited by Ehrenberg (1854), sample 1102]

***Amphora gracilis*** Ehrenb., Abh. Königl. Akad. Wiss. Berlin, 1841: 410, 1843. [cited by Ehrenberg (1854), sample 1106]

***Amphora libyca*** Ehrenb., Ber. Bekanntm. Verh. Königl. Preuss. Akad. Wiss. Berlin, 1840: 205, 1840. [cited by Ehrenberg (1854), 1085, Tefé Lake, samples 1099, 1102]

***Bacillaria australis*** Ehrenb.,Mikrogeologie, Atlas 8, Tafel XXXV-A, Fig II: 3, 1854. [cited by Ehrenberg (1854), sample 1085]

***Cocconeis lineata*** Ehrenb., Mikrogeologie, Atlas 8, Tafel XXXIX, Fig III: 11, 1854. [cited by Ehrenberg (1851), samples 1099, 1100]

***Cocconeis placentula*** Ehrenb., Infusionsthierchen, 194, 1838. [cited by Ehrenberg (1851, 1854), samples 1099)

***Cocconeis striata*** Ehrenb., Abh. Königl. Akad. Berlin, 1841: 370, Tafel III, Fig I: 30, 1843. [cited by Ehrenberg (1854), samples 1101, 1102].

***Cocconema gracile*** Ehrenb. Abh. Königl. Akad. Wiss. Berlin, 1841: 412, 1843. [cited by Ehrenberg (1851, 1854), sample 1099]

***Discoplea comta*** Ehrenb., Ber. Bekanntm. Verh. Königl. Preuss. Akad. Wiss. Berlin, 1844: 267. 1844. (nom. gen. inval.) [cited by Ehrenberg (1854), sample 1102]

***Eunotia amphioxys*** Ehrenb., Abh. Königl. Akad. Wiss. Berlin, 1841: 413, 1843. [cited by Ehrenberg (1851, 1854), samples 1099, 1102, 1106]

***Eunotia camelus*** Ehrenb., Abh. Königl. Akad. Wiss. Berlin, 1841: 413, 1843. [cited by Ehrenberg (1854), samples 1085, 1105]

***Eunotia diodon*** Ehrenb., Ber. Bekanntm. Verh. Königl. Preuss. Akad. Wiss. Berlin, 1837: 45, 1837. [cited by Ehrenberg (1851, 1854), samples 1099]

***Eunotia monodon*** Ehrenb., Abh. Königl. Akad. Wiss. Berlin, 1841: 414, 1843. [cited by Ehrenberg (1854), samples 1102]

***Eunotia parallela*** Ehrenb., Abh. Königl. Akad. Wiss. Berlin, 1841: 414, 1843. [cited by Ehrenberg (1854), sample 1102]

***Eunotia pileus*** Ehrenb., Abh. Königl. Akad. Wiss. Berlin, 1841: 414, 1843. [cited by Ehrenberg (1854), sample 1085]

***Eunotia praerupta*** Ehrenb., Abh. Königl. Akad. Wiss. Berlin, 1841: 414, 1843. [cited by Ehrenberg, (1854), Tefé Lake]

***Eunotia quaternaria*** Ehrenb., Abh. Königl. Akad. Wiss. Berlin, 1841: 414, 1843. [cited by Ehrenberg (1854), sample 1105]

***Eunotia nonaria*** Ehrenb. *ex* Rabenh., Fl. Eur. Alg., I, 71, 1864. [name already cited by Ehrenberg (1851), sample 1099]

***Eunotia octonaria*** Ehrenb. *ex* Rabenh., Fl. Eur. Alg., I, 71, 1864. [name already cited by Ehrenberg (1851), sample 1099]

***Eunotia quinaria*** Ehrenb., Abh. Königl. Akad. Wiss. Berlin, 1841: 414, 1843. [cited by Ehrenberg (1854), sample 1099]

***Eunotia senaria*** Ehrenb. *ex* Rabenh., Fl. Eur. Alg., I, 71, 1864. [name already cited by Ehrenberg (1851, 1854), sample 1099]

***Eunotia sphaerula*** Ehrenb., Abh. Königl. Akad. Wiss. Berlin, 1870: 55, 1870. [name already cited by Ehrenberg (1854), Tefé Lake, sample 1102]

***Eunotia tetraodon*** Ehrenb., Infusionsthierchen, 192, 1838. [cited by Ehrenberg (1854), sample 1085]

***Eunotia tridentula*** Ehrenb., Abh. Königl. Akad. Wiss. Berlin, 1841: 414, 1843. [cited by Ehrenberg (1841, 1854), sample 1099]

***Eunotia turgida***(Ehrenb.) Ehrenb., Infusionsthierchen, 190, 1838. [cited by Ehrenberg (1843), sample 1087]

***Fragilaria acuta*** Ehrenb., Ber. Bekanntm. Verh. Königl. Preuss. Akad. Wiss. Berlin, 1840: 210, 1840. [cited by Ehrenberg (1854), samples 1099, 1102]

***Fragilaria entomon*** Ehrenb., Abh. Königl. Akad. Wiss. Berlin, 1841: 415, 1843. [cited by Ehrenberg (1854), sample 1102]

***Gallionella crenata*** Ehrenb., Abh. Königl. Akad. Wiss. Berlin, 1847: 273, Tafel I Figs I: 8,9; Tafel I Figs II: 4,5; Tafel II Fig I: 12; Tafel II Figs II: 4,5; Tafel II Fig III: 3; Tafel III Fig I: 6; Tafel III Figs II: 9-11; Tafel IV Figs A: 8,9; Tafel IV, Tafel Figs B: 3-5; Tafel V Figs I: 5,6; Tafel VI Figs II: 4,6; 1849. [cited by Ehrenberg (1851, 1854), samples 1099, 1100]

***Gallionella distans*** Ehrenb., Infusionsthierchen, 190, 1838. [cited by Ehrenberg (1841, 1843, 1854), samples 1087?, 1104, 1105]

***Gallionella granulata*** Ehrenb., Abh. Königl. Akad. Wiss. Berlin, 1841: 415, 1843. [cited by Ehrenberg (1843, 1854), sample 1087?]

***Gallionella procera*** Ehrenb., Abh. Königl. Akad. Wiss. Berlin, 1847: 270, 273, 279, 285, 287, 293, 299, 303, 317, 319, 396, 399, 442, 443, 445, 447, 448, 449, 450, 451, 452, 453, 455, 456, 458, Tafel I Figs I: 6, 7; Tafel I Figs II: 3; Tafel II Figs I: 6, 7; Tafel II Figs II: 2, 3; Tafel II Fig III: 2; Tafel III Figs I: 3, 4; Tafel III Figs II: 4, 5; Tafel IV Figs A: 4, 5; Tafel IV Fig B: 2; Tafel V Fig I: 3; Tafel V Fig II: 4; Tafel VI Fig II:1. 1849. [cited by Ehrenberg (1854), Tefé Lake, sample 1085]

***Gallionella sulcata*** Ehrenb., Infusionsthierchen, 170, 1838. [cited by Ehrenberg (1854), sample 1102]

***Gallionella varians*** (C.Agardh)Ehrenb., Infusionsthierchen, 167, 1838. [cited by Ehrenberg (1851), sample 1099?]

***Gomphonema clavatum*** Ehrenb.,Abh. Königl. Akad. Wiss. Berlin, 1831: 88, 1832. [cited by Ehrenberg (1854), sample 1102]

***Gomphonema gracile*** Ehrenb.,Infusionsthierchen, 217, 1838. [cited by Ehrenberg (1851, 1854), samples 1099, 1100; 1102]

***Gomphonema longiceps*** Ehrenb. *ex* Ralfs, in Pritchard, Hist. Inf., ed. 4, 890, 1861. [name already cited by Ehrenberg (1854), sample 1085]

***Himantidium arcus***(Ehrenb.) Ehrenb., Ber. Bekanntm. Verh. Königl. Preuss. Akad. Wiss. Berlin, 1839: 127, 1839. [cited by Ehrenberg (1839, 1841, 1843, 1851, 1854), samples 1087, 1099, 1102, 1103, 1104, 1105]

***Himantidium gracile*** Ehrenb., Abh. Königl. Akad. Wiss. Berlin, 1841: 417, 1843. [cited by Ehrenberg (1854), samples 1099, 1101, 1102]

***Himantidium monodon*** Ehrenb.,Abh. Königl. Akad. Wiss. Berlin, 1841: 417, 1843. [cited by Ehrenberg (1851, 1854), sample 1099]

***Himantidium papilio*** Ehrenb.,Abh. Königl. Akad. Wiss. Berlin, 1841: 417, 1843. [cited by Ehrenberg (1854), sample 1085]

***Navicula amphioxys*** Ehrenb. Abh. Königl. Akad. Wiss. Berlin, 1841: 417, 1843. [cited by Ehrenberg (1841, 1843, 1854), samples 1087, 1102, 1104]

***Navicula amphisphenia*** Ehrenb.,Abh. Königl. Akad. Wiss. Berlin, 1841: 417, 1843. [cited by Ehrenberg (1854), 1085, Tefé Lake, samples 1102, 1104, 1106]

***Navicula bacillum*** Ehrenb., Abh. Königl. Akad. Wiss. Berlin, 1838: 130. 1839. [cited by Ehrenberg (1851, 1854), 1099, samples 1101, 1102]

***Navicula gracilis*** Ehrenb., Abh. Königl. Akad. Wiss. Berlin, 1831: 79, 1832. [cited by Ehrenberg (1854), sample from Tefé Lake, without number]

***Navicula sigma*** Ehrenb., Abh. Königl. Akad. Wiss. Berlin, 1833: 259, 1834. [cited by Ehrenberg (1854), sample 1102]

***Navicula silicula*** Ehrenb. Abh. Königl. Akad. Wiss. Berlin, 1841: 419, 1843. [cited by Ehrenberg (1854), sample from Tefé Lake, without number]

***Navicula viridis*** (Nitzsch)Ehrenb., Abh. Königl. Akad. Wiss. Berlin, 1831: 81, 1832. [cited by Ehrenberg (1841), sample 1088]

***Pinnularia amphirrhina*** Ehrenb., Mikrogeologie, Atlas 17, Tafel XV-A, Fig 20, 1854. [cited by Ehrenberg (1854), sample 1102]

***Pinnularia borealis*** Ehrenb. Abh. Königl. Akad. Wiss. Berlin, 1841: 420, 1843. [cited by Ehrenberg (1851, 1854), samples 1099, 1102]

***Pinnularia capitata***(Ehrenb.) Ehrenb., Ber. Bekanntm. Verh. Königl. Preuss. Akad. Wiss. Berlin, 1848: 18, 1848. [cited by Ehrenberg (1854), samples 1085, 1102]

***Pinnularia decurrens*** Ehrenb. Abh. Königl. Akad. Wiss. Berlin, 1841: 420, 1843. [cited by Ehrenberg (1851, 1854), samples 1085, 1099, 1102]

***Pinnularia dicephala***(Ehrenb.) Ehrenb. Abh. Königl. Akad. Wiss. Berlin, 1841: 420, 1843. [cited by Ehrenberg (1843, 1854), samples 1102, 1104]

***Pinnularia gastrum*** Ehrenb., Abh. Königl. Akad. Wiss. Berlin, 1841: 421, 1843. [cited by Ehrenberg (1854), samples 1099?, 1102?]

***Pinnularia gibba***(Ehrenb.) Ehrenb., Abh. Königl. Akad. Wiss. Berlin, 1841: 315, 1843. [cited by Ehrenberg (1854), sample 1102?]

***Pinnularia lanceolata***(Ehrenb.) Ehrenb., Abh. Königl. Akad. Wiss. Berlin, 1841: 315, 1843. [cited by Ehrenberg (1854), sample 1102]

***Pinnularia macilenta*** Ehrenb., Abh. Königl. Akad. Wiss. Berlin, 1841: 421, 1843. [cited by Ehrenberg (1854), samples 1102, 1104]

***Pinnularia nobilis*** (Ehrenb.) Ehrenb., Ber. Bekanntm. Verh. Königl. Preuss. Akad. Wiss. Berlin, 1845: 61, 1845. [cited by Ehrenberg (1843, 1854), sample 1104]

***Pinnularia tabellaria*** Ehrenb., Abh. Königl. Akad. Wiss. Berlin, 1841: 422, 1843. [cited by Ehrenberg (1851, 1854), sample 1099?]

***Pinnularia vespa*** Ehrenb., Mikrogeologie, pl. XXXIII, Fig V: 9, 1854. [name already cited by Ehrenberg (1851, 1854), sample 1099]

***Pinnularia viridis*** (Nitzsch) Ehrenb., Abh. Königl. Akad. Wiss. Berlin, 1841: 315, 1843. [cited by Ehrenberg (1843, 1851, 1854), samples 1099, 1100, 1102, 1104, 1105, 1106]

***Stauroneis gracilis*** Ehrenb., Abh. Königl. Akad. Berlin, 1841: 423, 1843. [cited by Ehrenberg (1854), sample 1099]

***Stauroneis phoenicenteron*** (Nitzsch) Ehrenb., Abh. Königl. Akad. Berlin, 1841: 311, 1843. [cited by Ehrenberg (1851, 1854), sample 1099]

***Stauroneis semen*** Ehrenb. *ex* Ralfs in Pritchard, His. Inf., ed. 4, 912, 1854. [cited by Ehrenberg (1851, 1854), samples 1099; 1101; from Tefé Lake?, without number]

***Stauroptera isostauron*** Ehrenb., Abh. Königl. Akad. Berlin, 1841: 423, 1843. [cited by Ehrenberg (1854), sample 1103?]

***Surirella bifrons*** Ehrenb., Abh. Königl. Akad. Berlin, 1833: 259, 1834. [cited by Ehrenberg (1854), sample 1102]

***Surirella constricta*** Ehrenb. *ex* Ralfs in Pritchard, Hist. Inf., ed. 4, 794, 1854. [cited by Ehrenberg (1854), samples 1102, 1103]

***Surirella euglypta*** Ehrenb., Abh. Königl. Akad. Berlin, 1841: 424, 1843. [cited by Ehrenberg (1851, 1854), samples 1099, 1102]

***Surirella oblonga*** Ehrenb. *ex* Ralfs in Pritchard, His. Inf., ed. 4, 795, 1854. [cited by Ehrenberg (1841, 1843, 1854), samples 1088, 1105]

***Synedra acuta*** Ehrenb. *ex* Ralfs in Pritchard, His. Inf., ed. 4, 788, 1854. [cited by Ehrenberg (1854), sample 1099]

***Synedra lunaris*** Ehrenb., Abh. Königl. Akad. Berlin, 1831: 87, 1832. [cited by Ehrenberg (1854), sample from Tefé Lake?, without number]

***Synedra ulna*** (Nitzsch) Ehrenb., Abh. Königl. Akad. Berlin, 1831: 87, 1832. [cited by Ehrenberg (1841, 1843, 1851, 1854), samples 1087, 1099, 1100?, 1104, 1105, 1106?, from Tefé Lake?, whitout number]

***Terpsinoe musica*** Ehrenb., Ber. Bekanntm. Verh. Königl. Preuss. Akad. Wiss. Berlin, 1843: 425, 1843. [cited by Ehrenberg (1854), sample 1102]

### Invalid names (unavailable proposals) or illegitimate names

As introduced before, Ehrenberg considered diatoms as “*animal poligastrica*” in all his publications. According to Article 45.4 of the ICBN, the validity of names of organisms originally not treated as plants, algae or fungi must be governed by the respective code (McNeill et al. 2006). Thus, the validity of names of diatoms published by Ehrenberg is ruled by the ICZN (Ride et al. 1999).

Article 12.1 of ICZN states that “to be available [valid], every new name published before 1931 must satisfy the provisions of Article 11 and must be accompanied by a description or a definition of the taxon that it denotes, or by an indication” (Ride et al. 1999). Subsequently, one of the meanings of the term “indication” and highlighted by us is the Article 12.2.7: “the proposal of a new genus-group name or of a new species-group name in association with an illustration of the taxon being named, or with a bibliographic reference to such an illustration”. Such conditions are not fulfilled by some names published by Christian Ehrenberg.

The names of diatoms listed below are those cited specifically by Ehrenberg (1841, 1843, 1851, 1854) from Brazilian samples and were considered by us as unavailable (invalid) (for cross code terms see David et al. 2012) because they do not fulfill Article 12 of the ICZN (Ride et al. 1999). On the other hand, in the case of *Navicula obtusa* Ehrenb. the name is available (valid), but illegitimate because it is a later homonym.

***Achnanthes brasiliensis*** Ehrenb., Mikrogeologie, 310, 311 1854. (nom. inval.)

***Achnanthes incrassata*** Ehrenb.**,** Mikrogeologie, 310, 311, 1854. (nom. inval.)

***Achnanthes ventricosa*** Ehrenb., Ber. Bekanntm. Verh. Königl. Preuss. Akad. Wiss. Berlin, 1848: 479, 1848. (nom. inval.)

***Cocconeis glans*** Ehrenb., Mikrogeologie, 311, 1854. (nom. inval.)

***Cocconeis lineata var. brasiliensis*** Ehrenb., Mikrogeologie, 309, 310, 311, 1854. (nom. inval.)

***Cocconeis lirata*** Ehrenb., Mikrogeologie, 311, 1854. (nom. inval.)

***Eunotia amphioxys* var. *rostrata*** Ehrenb., Ber. Bekanntm. Verh. Königl. Preuss. Akad. Wiss. Berlin, 1851: 228, 1851. (nom. inval.)

***Eunotia carinata*** Ehrenb., Ber. Bekanntm. Verh. Königl. Preuss. Akad. Wiss. Berlin, 1851: 477, 1851. (nom. inval.)

***Eunotia denaria*** Ehrenb., Ber. Bekanntm. Verh. Königl. Preuss. Akad. Wiss. Berlin, 1851: 477, 1851 (nom. inval.)

***Eunotia edulis*** Ehrenb., Mikrogeologie, 315-650, 1854. (nom. inval.)

***Eunotia paradoxa*** Ehrenb., Ber. Bekanntm. Verh. Königl. Preuss. Akad. Wiss. Berlin, 1843: 139, 1843. (nom. inval.) non *Eunotia paradoxa* Berg., Bot. Not. 1939: 433, 1939.

***Eunotia triglyphis*** Ehrenb., Ber. Bekanntm. Verh. Königl. Preuss. Akad. Wiss. Berlin, 1851: 228, 1851. (nom. inval.)

***Fragilaria australis*** Ehrenb., Mikrog eologie, 308, 311, 1854. (nom. inval.)

***Gallionella tenerrima*** Ehrenb., Ber. Bekanntm. Verh. Königl. Preuss. Akad. Wiss. Berlin, 1842: 272, 1842. (nom. inval.)

***Navicula catharinae*** Ehrenb., Mikrogeologie, 310, 311, 1854. (nom. inval.)

***Navicula microstauron*** Ehrenb., Ber. Bekanntm. Verh. Königl. Preuss. Akad. Wiss. Berlin, 1841: 203, 1841. (nom. inval.)

***Navicula obtusa*** Ehrenb.,Abh. Königl. Akad. Berlin, , 1841: 419, 1843. (nom. illeg.) *non Navicula obtusa* Bory, Encycl. Méth., Hist. Nat. Zooph., 563, 1827. *nec Navicula obtusa* (Lagerst.) Hust., Süssw.-Diat. Deutschl., 36. 1909 (nom. illeg.)

***Pinnularia catharinae*** Ehrenb., Mikrogeologie, 311, 1854. (nom. inval.)

***Pinnularia decora*** Ehrenb., Mikrogeologie, 311, 1854. (nom. inval.)

***Pinnularia digitus*** Ehrenb., Ber. Bekanntm. Verh. Königl. Preuss. Akad. Wiss. Berlin, 1845: 61, 1845. (nom. inval.)

***Pinnularia formica*** Ehrenb.,Mikrogeologie, 311, 1854. (nom. inval.) *non Pinnularia formica* (Ehrenb.) R.M.Patrick, Monogr. Acad. Nat. Sc. Philad., 13, 627, 1966.

***Raphoneis laevigata*** Ehrenb., Mikrogeologie, 310, 311, 1854. (nom. inval.)

***Stauroptera brasiliensis*** Ehrenb., Mikrogeologie, 311, 1854. (nom. inval.)

***Surirella ovata*** Ehrenb., Ber. Bekanntm. Verh. Königl. Preuss. Akad. Wiss. Berlin, 1844: 341, 1844. (nom. inval.)

## Discussion

Biological collections represent a set of organism that lived in some locality during some time. Thus, historical collections are a portrait of the biodiversity of some place that can never be faithfully restudied. This is due to natural or anthropogenic factors responsible for changes of biological diversity of an environment in short, medium or long terms, (Jackson and Sax 2009). Therefore, historical collections of species are indispensable sources to study the biodiversity on earth (Shaffer et al. 1998).

Besides their scientific importance, such collections also perform a fundamental cultural role regarding the region in which the material was collected and for the development of science along the centuries. Through Ehrenberg’s studies, for instance, it can be inferred which pathway some botanists such as Carl Friedrich Philipp von Martius, Karl Sigismund Kunth, Eduard Friedrich Poeppig, Édouard Louis Chavannes, and other personalities such as the Carl Pabst or Hermann Encke, took when traveling Brazil in the 19^th^ century.

The cultural and scientific importance of the Brazilian samples studied by Ehrenberg can be found in its historical context. At the beginning of the 19^th^ century, many regions in Brazil were occupied by natives whose social organization was different of other native people from South America. In 1818, Prince Regent D. Joao VI of Portugal started an immigration policy that resulted in the arrival of 1,458 Swiss immigrants between 1819 and 1820. These immigrants established themselves on the Cantagalo Farm and this settlement was known as Nova Friburgo. In 1824, Nova Friburgo received about 450 new people, this time German immigrants. Thus, it can be estimated that approximately 1,900 people lived in this region. In 1907, the Instituto Brasileiro de Geografia e Estatística already estimated about 19,185 inhabitants and in 2011 there were estimates of 182,748 inhabitants (IBGE 1912, 2011). In between, in 1850, Hermann Encke sent two samples to Ehrenberg.

Although we have no accurate estimates of the population from Nova Friburgo in 1850, it is clear that the impact on biodiversity generated by less than 19,185 inhabitants (in 1907) is lower than the impact of the current 182,748 inhabitants. This example can be translated to the other samples collected in Brazil until the first half of the 19^th^ century. Even if it refers to only few places, these materials may be the most accurate examples of the biodiversity of Brazilian diatoms. Moreover, historical samples like those can help to understand anthropogenic effects on the biodiversity of diatoms in tropical and subtropical regions.

The list of Brazilian diatoms published in Ehrenberg’s studies allowed also to discuss the taxonomy and nomenclature of some diatoms described in the 19^th^ century. Although the taxonomy of the diatoms published by Ehrenberg are ruled by the ICZN, the conditions required for a taxon to be considered available (valid) are practically the same as those of the ICBN, that is, a description, a definition (differential diagnosis) or an indication (which can be a published illustration) are needed. This last issue justifies, for instance, to ensure the availability of species such as *Bacillaria australis*, *Gallionella crenata*, *Gallionella procera*, *Pinnularia amphirrhina* and *Pinnularia vespa* which have no diagnosis, but indications to an illustration was provided.

Some unavailable names cited by Ehrenberg continued to be ruled by the ICZN which were available by him in later studies. This happened to *Eunotia sphaerula* and *Pinnularia vespa* which were cited by Ehrenberg (1854) and Ehrenberg (1851), respectively, but were made available by Ehrenberg (1870) and Ehrenberg (1854), respectively. Other species cited by Ehrenberg from Brazilian samples became ruled by the ICBN when validated by authors who treated diatoms as algae. This was the case with *Eunotia nonaria*, *Eunotia octonaria* and *Eunotia senaria*, validated by Rabenhorst (1864), and *Gallionella longiceps* validated by Ralfs (Pritchard 1861). Internal evidences enabled us to identify the author of the taxa by “Ehrenb. *ex*”, linking them to the types from the Ehrenberg material.

Similarly, *Navicula obtusa* Ehrenb.(non *Navicula obtusa* Bory), the only illegitimate name among the 101 names published by Ehrenberg from Brazilian samples, was legitimated by Rabenhorst (1864, p. 197) with the new name *Navicula appendiculata* f. *obtusa*. This taxon was associated to “Ehrenb. (Verb. p. 131)”. The term “Verb.” mentioned by Rabenhorst (1864) referred to the paper *Verbreitung und Einfluss des mikroskopischen Lebens in Süd- und Nord-Amerika*, published by Ehrenberg (1843). The paper that we had access has 154 pages, betweem the page 291 to 445 and, therefore, Rabenhorst (1864) referred to a page that does not exist on the paper that we analyzed, but maybe referes to a reprint version that we had not access. A similar fact is seen with *Eunotia bidens* Ehrenb. and *Gomphonema cygnus* Ehrenb. which were cited by Rabenhorst (1864, p. 74 and 286, respectively) and related to “Ehrenb. (Verb. p. 125 …)” and “Ehrenb. (Verb. p. 128 …)”. The difference between the pages cited by Rabenhorst (1864) and the correct page on Ehrenberg (1843) in both cases is 288 pages. Therefore, we can tell that Rabenhorst (1864) associated *Navicula appendiculata* f. *obtusa* with *Navicula obtusa* in Ehrenberg (1843, p. 419), the only taxa with the epithet *obtusa* on the page 419, even though he had not mentioned clearly *Navicula obtusa*.

According to the ICBN, an epithet of a latter homonym can be used in a different combination and on a different rank, if the epithet is available at this rank (Article 58.1, McNeill et al. 2006). In this case, the name is treated as new. Thus, the authorship published by Rabenhorst (1864) should be *Navicula appendiculata* f. *obtusa* Rabenh. and not *Navicula appendiculata* f. *obtusa* (Ehrenb.) Rabenh., which is in agreement with the Example 1 of the Article 58.1 of the ICBN (McNeill et al. 2006).

Another nomenclatural issue is about valid descriptions or definitions, which we have considered to define valid publication of some diatoms published by Ehrenberg. Some taxa published in the 19^th^ century showed very short descriptions or diagnoses. This led us to consider the expression “…*und* Terpsinoë brasiliensis, *mit sehr kleinen Notenzeichen*…” provided by Ehrenberg (1854) as definition (differential diagnosis) of *Terpsinoe brasiliensis* in relation to *Terpsinoe musica*.

On the other hand, species such as *Navicula gracilis*, cited by Ehrenberg (1854) for Tefé Lake, and other species such as *Gomphonema discolor*, *Navicula fusiformis*, *Navicula gibba*, *Navicula turgida*, and *Navicula uncinata*, which were not cited for Brazil, showed one particularity. They were defined initially by Ehrenberg (1832a) by only a range of measures of specimens from different localities and can thus not be considered a valid description. According to the ICZN, description is “a statement in words” (Ride et al., 1999) and, therefore, only measurements do not constitute a description of some taxon. Thereby, *Navicula gracilis* as well as *Gomphonema discolor*, *Navicula fusiformis*, *Navicula gibba*, *Navicula turgida* and *Navicula uncinata* have to be considered available (valid) only according to Ehrenberg (1832b), when they were provided with a valid description. This implies that the attribution of the author of *Navicula gracilis* to Ehrenberg (1832a), as found in Algaebase (Guiry & Guiry, 2012) is not correct and should be changed to Ehrenberg (1832b).

Despite similarities among the ICZN and ICBN of a valid name, one difference must be highlighted: the independence of the validity of the name of the genus and the name of the species. In the ICZN, a species name is available even if the genus name is not available (Article 11.9.3.1, Ride et al. 1999) while according to the ICBN this condition is not allowed (Article 43.1, McNeill et al. 2006). This refers to *Discoplea comta* (Silva 2003), a name cited for Brazil. However, we notice problems about the original sense of this species and its concept (Silva 2003, Kusber and Jahn 2009).

Nagumo (2003) also noticed a similar condition concerning *Amphora libyca* when carrying out lectotypification of this species. Thus, the two cases showed that the names listed by Ehrenberg for Brazil do not necessarily correspond to the current concept of the taxa and highlight the need for future studies that reinvestigate the preparations and/or samples from Brazil and compare the types with the names cited according to Ehrenberg’ concept.

Even considering these limitations, it is noteworthy that only about 43% of the valid names recorded by Ehrenberg (1839, 1841, 1843, 1851, 1854) and compiled here were reported for Brazil by other researches. The other 57% include taxa of *Eunotia*, *Pinnularia* and other genera that even under their synonyms were not recorded for Brazil, which corresponds to 44 names. The 77 diatom names cited by Ehrenberg (1839, 1841, 1843, 1851, 1854) make him the most important diatomologist of Brazilian diatoms in the first half of the 19^th^ century.

## Conclusion

We catalogued 101 taxa recorded by Christian Gottfried Ehrenberg in a set of five studies, among which 77 are available (valid) names, 24 names are unavailable (invalid) and one is illegitimate. The reason why these 24 names are considered as invalid is the absence of a description or a definition (differential diagnosis) or the indication of an illustration. Among the 77 valid names, five were originally described for Brazil and are here lectotypified. Only 34 taxa of this list had been recorded until now by other studies than Ehrenberg’s. The other 57% of the valid taxa can be treated as first citation of the name for the Brazilian diatom flora.

In the future, studies should be conducted to characterize all specimens of Brazilian diatoms described by Ehrenberg. This could be carried out through the sampling and analysis of recent material from the original locality of the lectotype.

With this compilation, we can go back to the Ehrenberg collection to compare Ehrenberg’s identification as well as the current and the original concept of the species of diatoms from Brazil recorded by him. For this, studies with new preparations from his original samples will be necessary. Finally, it will be possible to carry out new studies comparing the old and recent diatom flora and associate the likely changes with the historical variables. This could provide tools to understand changing mechanisms of the biodiversity of diatoms in tropical and subtropical habitats.

## Supplementary Material

XML Treatment for Eunotia bidens

XML Treatment for Eunotia depressa

XML Treatment for Eunotia elephas

XML Treatment for Pinnularia microstauron

XML Treatment for Terpsinoe brasiliensis
